# Post-synaptic scaffold protein TANC2 in psychiatric and somatic disease risk

**DOI:** 10.1242/dmm.049205

**Published:** 2022-03-04

**Authors:** Lillian Garrett, Patricia Da Silva-Buttkus, Birgit Rathkolb, Raffaele Gerlini, Lore Becker, Adrian Sanz-Moreno, Claudia Seisenberger, Annemarie Zimprich, Antonio Aguilar-Pimentel, Oana V. Amarie, Yi-Li Cho, Markus Kraiger, Nadine Spielmann, Julia Calzada-Wack, Susan Marschall, Dirk Busch, Carsten Schmitt-Weber, Eckhard Wolf, Wolfgang Wurst, Helmut Fuchs, Valerie Gailus-Durner, Sabine M. Hölter, Martin Hrabě de Angelis

**Affiliations:** 1 Institute of Experimental Genetics and German Mouse Clinic, Helmholtz Zentrum München, German Research Center for Environmental Health, 85764 Neuherberg, Germany; 2 Institute of Developmental Genetics, Helmholtz Zentrum München, German Research Center for Environmental Health, 85764 Neuherberg, Germany; 3 German Center for Diabetes Research (DZD), Helmholtz Zentrum München Ingolstädter Landstr. 1, 85764 Neuherberg, Germany; 4 Institute of Molecular Animal Breeding and Biotechnology, Gene Center, Ludwig-Maximilians University Munich, 81377 Munich, Germany; 5 TUM School of Life Sciences, Technische Universität München, 85354 Freising-Weihenstephan, Germany; 6 Institute for Medical Microbiology, Immunology and Hygiene, Technische Universität München, Trogerstrasse 30, 81675 Munich, Germany; 7 Center of Allergy and Environment (ZAUM), Technische Universität München, and Helmholtz Zentrum München, 85764 Neuherberg, Germany; 8 Chair of Developmental Genetics, TUM School of Life Sciences, Technische Universität München, 85354 Freising-Weihenstephan, Germany; 9 Deutsches Institut für Neurodegenerative Erkrankungen (DZNE) Site Munich, Feodor-Lynen-Str. 17, 81377 Munich, Germany; 10 Munich Cluster for Systems Neurology (SyNergy), Adolf-Butenandt-Institut, Ludwig-Maximilians-Universität München, Feodor-Lynen-Str. 17, 81377 Munich, Germany; 11 Chair of Experimental Genetics, TUM School of Life Sciences, Technische Universität München, Alte Akademie 8, 85354 Freising, Germany

**Keywords:** TANC2, Neurodevelopmental disorder, Somatic comorbidity, Mouse models

## Abstract

Understanding the shared genetic aetiology of psychiatric and medical comorbidity in neurodevelopmental disorders (NDDs) could improve patient diagnosis, stratification and treatment options. Rare tetratricopeptide repeat, ankyrin repeat and coiled-coil containing 2 (*TANC2*)-disrupting variants were disease causing in NDD patients. The post-synaptic scaffold protein TANC2 is essential for dendrite formation in synaptic plasticity and plays an unclarified but critical role in development. We here report a novel homozygous-viable *Tanc2*-disrupted function model in which mutant mice were hyperactive and had impaired sensorimotor gating consistent with NDD patient psychiatric endophenotypes. Yet, a multi-systemic analysis revealed the pleiotropic effects of *Tanc2* outside the brain, such as growth failure and hepatocellular damage. This was associated with aberrant liver function including altered hepatocellular metabolism. Integrative analysis indicates that these disrupted *Tanc2* systemic effects relate to interaction with Hippo developmental signalling pathway proteins and will increase the risk for comorbid somatic disease. This highlights how NDD gene pleiotropy can augment medical comorbidity susceptibility, underscoring the benefit of holistic NDD patient diagnosis and treatment for which large-scale preclinical functional genomics can provide complementary pleiotropic gene function information.

## Abstract (precis)

**Summary:** Disruption of mouse *Tanc2* causes brain and liver abnormality, increasing psychiatric and somatic disease risk long term, highlighting the benefit of holistic diagnosis and treatment approaches for human neurodevelopmental disorder.

## INTRODUCTION

Neurodevelopmental disorders (NDDs), including intellectual disability and autism spectrum disorder (ASD), represent a significant personal and societal burden [∼15% of the US population (Zablotsky et al., 2019; American Psychiatric Association, 2013)]. The symptoms of these complex diseases overlap, implying a common genetic underpinning. For instance, high-risk NDD gene mutations disrupt convergent molecular pathways involved in synaptic strength and plasticity (Cross-Disorder Group of the Psychiatric Genomics Consortium. Electronic address and Cross-Disorder Group of the Psychiatric Genomics, 2019). As well as neuropsychiatric disturbance, NDD patients are at higher risk of medical comorbidity for which routine clinical screening is not standard (Tye et al., 2018). Thus, understanding the pathogenesis and genetic aetiology of associated systemic abnormality and somatic disease risk could have important implications not just for NDD patient life quality but also for diagnosis and patient stratification in precision treatment strategies.

Tetratricopeptide repeat, ankyrin repeat and coiled-coil containing 2 (*TANC2*), located at chromosome 17q23, is one such high-risk NDD candidate gene (Guo et al., 2019). It encodes a large 200-kDa TANC2 protein that consists of 1990 amino acid residues. Named after its domain architecture, it comprises tetratricopeptide (TPR) and ankyrin repeats (ANK), a coiled-coil domain as well as a C-terminal PDZ-interacting motif. It is widely expressed in the developing human (in excitatory neurons and radial glial cells) and rodent brain as well as in the adult brain as a scaffold in the postsynaptic density (PSD) of excitatory neurons. As such, it facilitates cell signalling downstream of cell surface glutamate receptors through multi-protein complex formation with other PSD proteins (e.g. PSD95, SHANK1), influencing dendritic spine formation and synaptic strength (Suzuki et al., 2005).

Recently, rare *de novo* and inherited disruptive *TANC2* mutations (including 16 *TANC2*-truncating variants) were disease causing in 20 patients with NDD syndrome (Guo et al., 2019). The majority of these mutations occurred before the C-terminal PDZ-interacting motif that is essential for dendritic localisation of TANC2. Although this indicates a critical role for *TANC2* in brain development and NDD pathogenesis, the gene likely has pleiotropic effects given its expression in organs outside the brain (https://www.proteinatlas.org/ENSG00000170921-TANC2/tissue). Preliminary evidence that *TANC2-*disrupted NDD patients exhibit systemic abnormalities, including craniofacial dysmorphology, supports this assertion (Guo et al., 2019). Moreover, the myriad TANC2 interaction partners necessarily implicate this protein in multiple developmental and adult-based functions (Gasparini et al., 2017). Nevertheless, a detailed understanding of these peripheral manifestations, as well as their long-term consequence for patients, are lacking.

One approach to crystallise the genetic intersection of NDDs and somatic disease pathogenesis is to leverage preclinical functional genomic information such as that from the International Mouse Phenotyping Consortium (IMPC). The IMPC endeavours to functionally annotate all protein-coding genes and render the multi-systemic phenomic information freely available (see www.mousephenotype.org, 7824 genes to date; Brown and Moore, 2012). Importantly, the implementation of mouse phenotype (MP) ontology terms (Smith and Eppig, 2009) permits integrative and comparative approaches to understand mutation effects translatable to humans. It is thus a useful tool to identify the brain-derived and somatic manifestations associated with disease-causing NDD genes such as *Tanc2*.

With a focus on *Tanc2* pleiotropy, we here illustrate how the IMPC database can probe in depth the shared genetic risk for psychiatric and non-psychiatric disease associated with a high-risk NDD gene. A previous *Tanc2* disruption model, made using Genetrap technology, was homozygous lethal, confirming the critical role of TANC2 in embryogenesis (Han et al., 2010). The homozygous *Tanc2* disruption model that we generated here for IMPC using CRISPR/Cas9-mediated targeted mutagenesis (*Tanc2^-em1/CRISPR/Cas^*) was viable and allowed us to assess the function of this gene beyond development into adulthood. In young adults, we performed a comprehensive multi-systemic characterisation, revealing a disrupted TANC2-related syndrome with potential translational relevance for patients with rare biallelic *TANC2* variants and autosomal-recessive NDD. With integrative analysis, we identified psychiatric and systemic abnormality covariance to elaborate potential multimodal NDD biomarkers and the likely underlying developmental signalling pathway obstruction. Our findings illustrate how preclinical data can evince the shared genetic aetiology between psychiatric and somatic disease, potentially harnessing the latter to improve diagnosis and treatment prospects.

## RESULTS

### *Tanc2* disruption induces hyperactivity and impairs sensorimotor gating

**Fig. 1.**
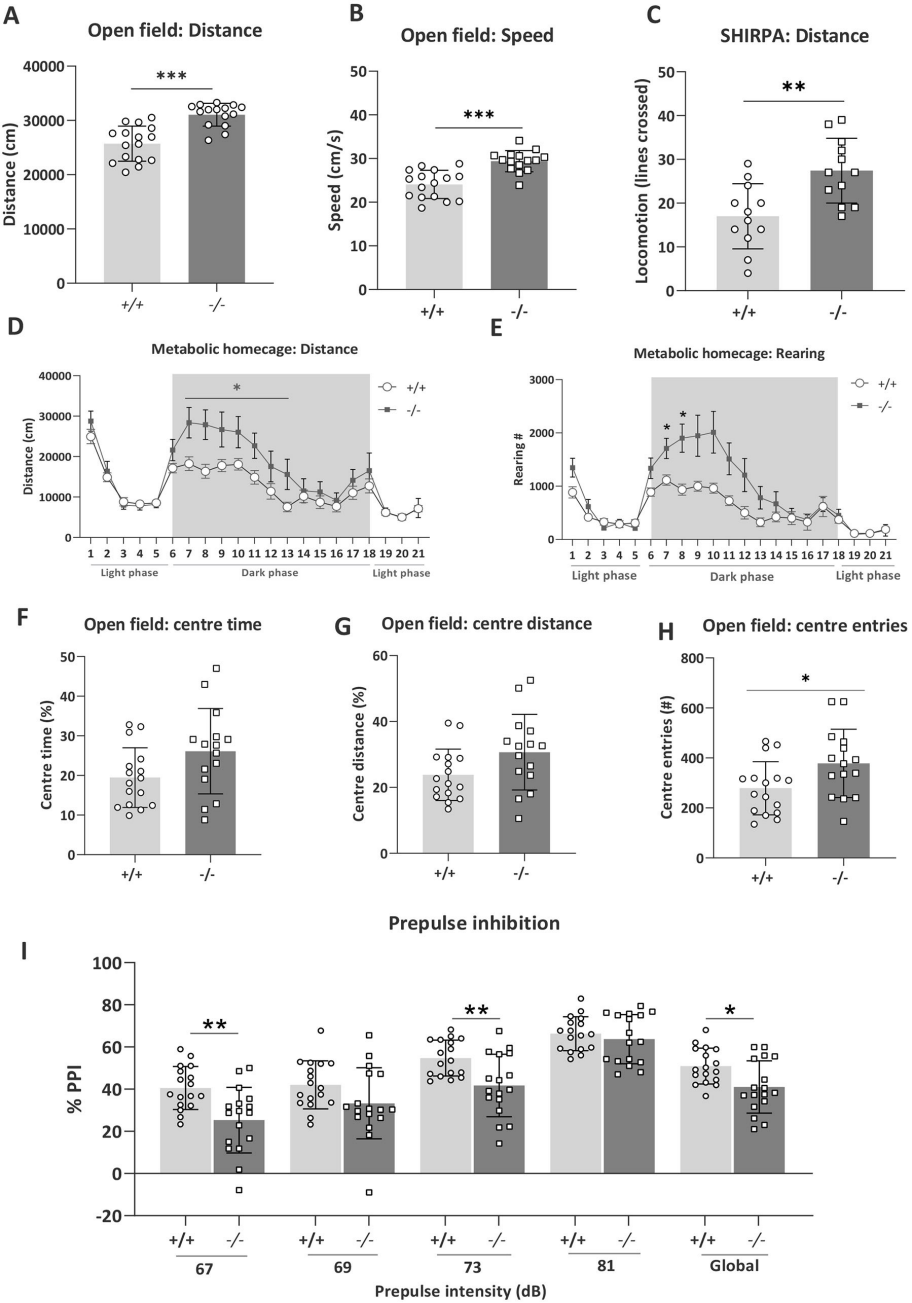
**Disruption of *Tanc2* causes hyperactivity in both novel and homecage environments and impairs sensorimotor gating.** (A-E) *Tanc2*^−/−^ mutant mice were clearly hyperactive in response to a novel open field [increased distance travelled (A) and speed (B)] and SHIRPA [increased lines crossed (C)] environment and showed both increased distance travelled (D) and rearing activity (E) during the dark phase while housed in the metabolic cages for 21 h. Shaded areas indicate dark phase, 18:00-06:00. (F-H) *Tanc2*^−/−^ mutant mice showed a pattern of increased % centre time (F), % centre distance (G) and centre entries (H) in open field, suggesting a slight anxiolytic effect. (I) % Prepulse inhibition (PPI) was also decreased in the mutant mice and significant at 67 dB and 73 dB prepulse (PP) intensities and global, the mean of all four prepulses. The PPI deficit was not evident at the 81 dB PP intensity [16 dB above background noise (65 dB)]. *P<0.05, **P<0.01, ***P<0.001 +/+ versus −/−, males and females pooled (unpaired Student's *t*-test) (see Table S1 for sex and genotype group numbers for each test). Data are mean±s.d.

We observed that *Tanc2* disruption induced hyperactivity evident across multiple contexts. In response to the mild novelty stress of open field (OF) and SHIRPA, the *Tanc2*^−/−^ mice showed heightened locomotor responses [MP: 0001399, 0003313; OF increased distance travelled and speed (unpaired Student's *t*-test: distance, *t*(29)=5.40, *P*<0.0001; speed, *t*(29)=5.17, *P*<0.0001; Fig. 1A,B] and SHIRPA increased lines crossed [unpaired Student's *t*-test: lines crossed, *t*(22)=3.45, *P*=0.0023; Fig. 1C)]. For distance travelled in the OF, we also observed that the effect tended to be more pronounced in males (Fig. S4). In the metabolic homecages (MHCs), *Tanc2* disruption induced increased levels of both locomotor (distance) and exploratory (rearing) activity during the dark phase, reaching significance during the first hours after lights off [repeated measures (RM) ANOVA genotype effect: *F*(1,31)=4.93, *P*=0.03 (distance, 7-13h), genotype × time interaction *F*(6,198)=2.59, *P*=0.02 (rearing, 7-13 h); Fig. 1D,E]. Thus, the hyperactivity consequent to *Tanc2* interference occurs across multiple contexts, suggesting a global propensity to elevated activity.

In terms of anxiety-related behaviour in the OF, there was a pattern of increased % centre time (Fig. 1F), % centre distance (Fig. 1G) and centre entries (Fig. 1H) [unpaired Student's *t*-test: centre time, *t*(29)=2.01, *P*=0.05; centre distance, *t*(29)=1.95, *P*=0.06; centre entries, *t*(29)=2.27, *P*=0.03]. Together, this indicates a slight anxiolytic effect of *Tanc2* disruption, but should be interpreted with caution given the pronounced hyperactivity of the mice potentially influencing the amount of time and activity in the central more aversive zone of the OF.

Prepulse inhibition (PPI) has been described in mice and humans as an operational measure of sensorimotor gating, reflecting the ability to successfully integrate and inhibit sensory information (Geyer et al., 2002). The loss of *Tanc2* impaired % PPI (Fig. 1I; MP: 0009142). This difference was significant at the 67 dB [unpaired Student's *t*-test: *t*(32)=3.37, *P*=0.002] and 73 dB [unpaired Student's *t*-test: *t*(32)=3.13, *P*=0.004] prepulse (PP) intensities and at global [mean response to all PPs, unpaired Student's *t*-test: *t*(32)=2.71, *P*=0.01]. There was a tendency to impairment at 69 dB PP intensity [unpaired Student's *t*-test: *t*(32)=1.78, *P*=0.08] with no difference at the 81 dB PP intensity (16 dB above background). This indicates a specific deficit in sensorimotor gating ability. We did not observe differences in either the acoustic startle or auditory brainstem responses, thus excluding alterations in hearing ability and neuromuscular recruitment (Fig. S5A,B). Furthermore, in spite of the role of TANC2 in neurodevelopment and differences in body weight (see below), in adult −/− mice, we did not observe obvious differences in brain size between the genotypes (Fig. S3; MP: 0000774; decreased brain size, no difference; MP: 0005238, increased brain size, no difference).

### *Tanc2* disruption lowers body weight and alters indices of metabolic function

**Fig. 2.**
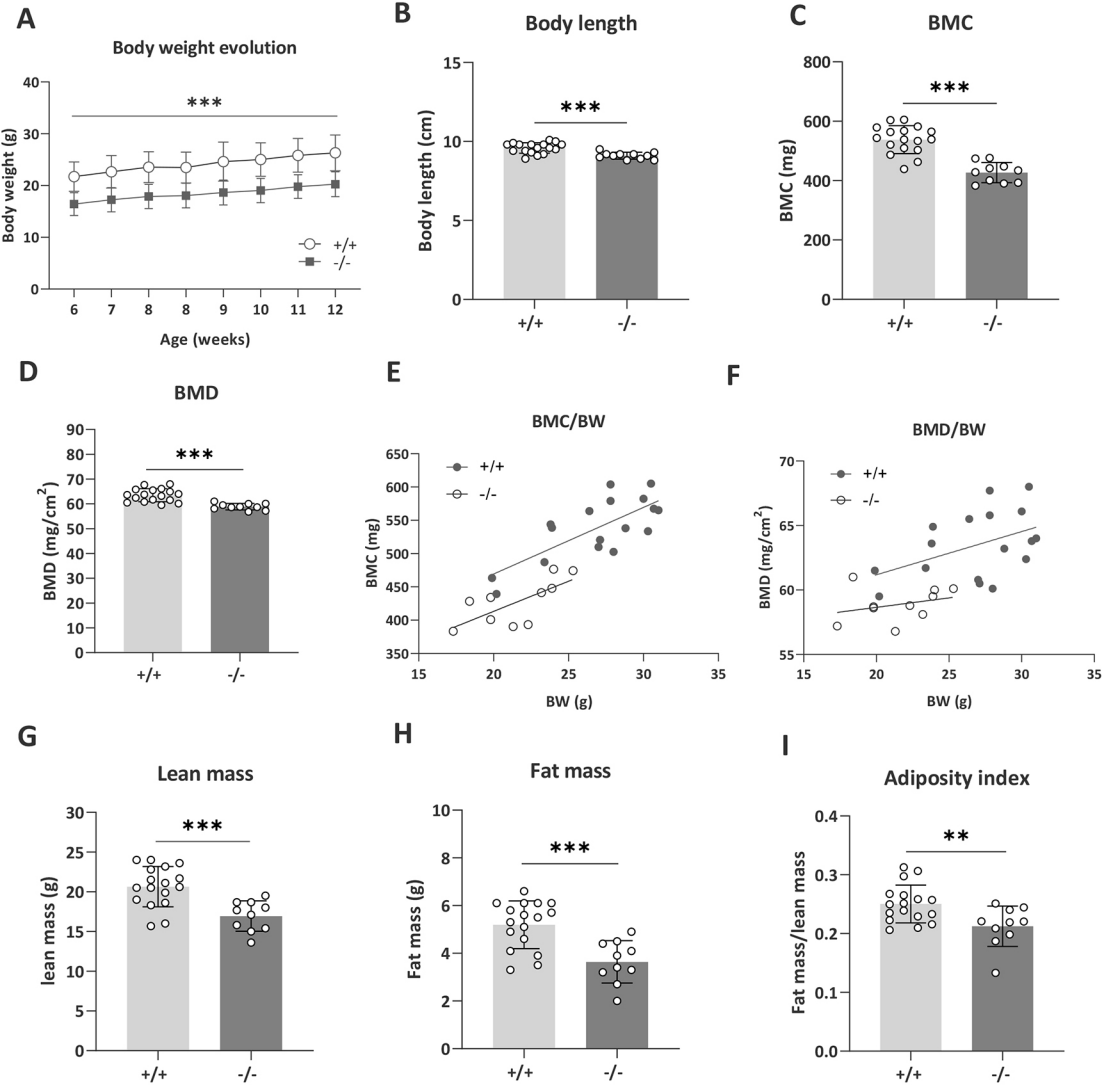
***Tanc2* disruption alters body size and adipose tissue distribution.** (A) *Tanc2*^−/−^ mice showed decreased body weight at all time points over the course of the analysis period, yet no difference in body weight gain was detected. (B-E) Body length (B), bone mineral content (BMC) (C) and bone mineral density (BMD) (D) were decreased, and BMC correlated positively with body weight (BW) (E). (F) BMD was not strongly predicted by BW. (G-I) Lean mass (G) and fat mass (H) were reduced, and there was a shift in body composition towards less fat in favour of lean mass, as indexed by decreased fat mass/lean mass ratio (I). Data are mean±s.d., males and females pooled. **P<0.01, ***P<0.001, +/+ versus −/− [linear mixed effect model (body weight), unpaired Student's *t*-test].

We measured body weight evolution to index mouse wellbeing. *Tanc2* disruption clearly decreased body weight at all time points [Fig. 2A; mixed effect analysis, fixed effects (type III) genotype *F*(1,33)=50.87, *P*<0.0001; MP: 0001262] with body weight gain comparable. The mutant mice were also of smaller stature, seen as decreased body length [Fig. 2B; unpaired Student's *t*-test: *t*(26)=4.30, *P*=0.0002; MP: 0001258]. Both bone mineral content (BMC) (Fig. 2C) and bone mineral density (BMD) (Fig. 2D) were decreased [unpaired Student's *t*-test: BMC, *t*(25)=6.52, *P*<0.0001, BMD, *t*(25)=5.18, *P*<0.0001]. BMC is likely to be related to the lower body size as it was predicted by body weight in linear regression (linear regression BMC: +/+, *R*^2^=0.56, *P*=0.0005; −/−, *R*^2^=0.49, *P*=0.02; Fig. 2E). BMD was not significantly predicted by body weight (Fig. 2F; BMD +/+, *R*^2^=0.20, *P*=0.07; −/−, *R*^2^=0.09, *P*=0.41; MP: 0000063). The lean mass [unpaired Student's *t*-test: *t*(25)=3.97, *P*=0.0005; Fig. 2G] and fat mass [unpaired Student's *t*-test: *t*(25)=4.06, *P*=0.0004; Fig. 2H] were both reduced. However, the adiposity index of fat mass relative to lean mass was also reduced, suggesting a body composition shift from decreased fat in favour of increased lean mass [Fig. 2I; unpaired Student's *t*-test: *t*(25)=2.89, *P*=0.008].

**Fig. 3.**
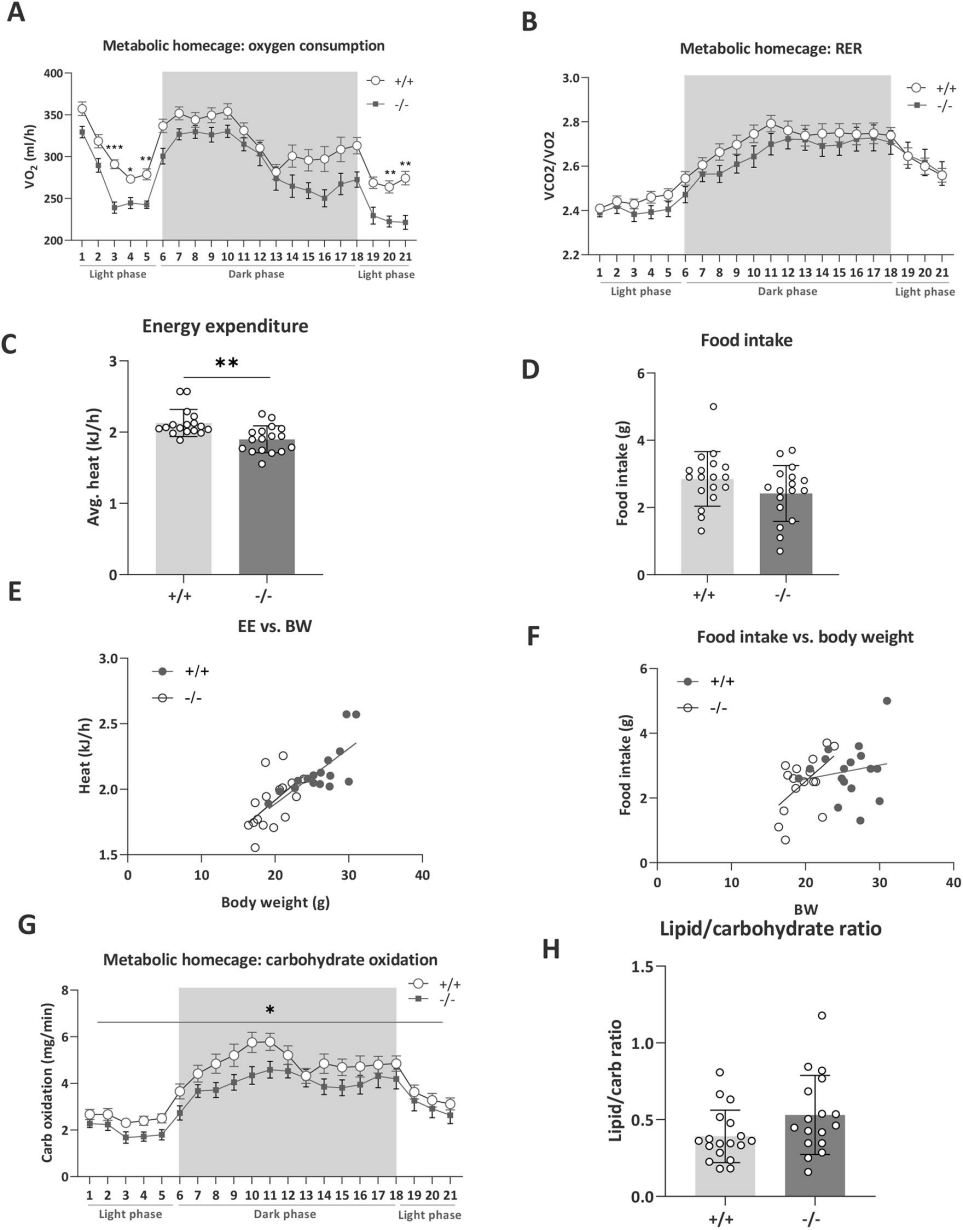
***Tanc2* disruption alters metabolic rate, food intake and substrate utilisation profiles.** (A,B) *Tanc2* disruption in mice leads to decreased oxygen consumption (VO_2_) during indirect calorimetry analysis in metabolic homecages (A), while respiratory exchange ratio (RER; VCO_2_/VO_2_) was not markedly altered but tended to decrease (B). (C) Furthermore, the metabolic rate, as indexed by energy expenditure in kJ/h, was significantly decreased. (D-F) *Tanc2* disruption led also to decreased food intake (D); however, both energy expenditure (EE; E) and food intake (F) were correlated with the lower body weight of the mice. (G,H) *Tanc2* disruption also caused an altered substrate utilisation profile in which carbohydrate oxidation (G) was decreased and more lipids were oxidised relative to carbohydrates, as shown in the increased lipid/carbohydrate oxidation ratio (H). *P<0.05, **P<0.01, ***P<0.001 +/+ versus −/− [unpaired Student's *t*-test or repeated measures (RM) ANOVA with post-hoc Sidak's test]. Grey shaded area demarcates the dark phase. Data are mean±s.d., males and females pooled.

In terms of metabolic processes, *Tanc2* disruption resulted in reduced oxygen consumption (VO_2_; MP: 0005290) in MHCs, particularly significantly during the inactive rather than the active phase [RM ANOVA, time×genotype interaction effect: *F*(20, 660)=1.73, *P*=0.03; see Fig. 3A for post-hoc significance]. The respiratory exchange ratio [RER; MP: 0010379, oxygen consumption relative to carbon dioxide production (VCO_2_/VO_2_)] was not strongly altered (Fig. 3B). Disruption of *Tanc2* decreased the metabolic rate of the mice [energy expenditure, unpaired Student's *t*-test: *t*(32)=3.51, *P*=0.001; Fig. 3C; MP: 0004890] that was predicted by body weight [simple linear regression: *R*^2^=0.54 (+/+), *P*=0.001, *R*^2^=0.31 (−/−), *P*=0.02; Fig. 3E]. Although a pattern of decreased food intake was evident (Fig. 3D; MP: 0011940), this also tended to correlate with body weight (Fig. 3F). Substrate utilisation profile was altered where carbohydrate oxidation decreased [mixed-effects analysis, genotype effect: *F*(1,33)=6.14, *P*=0.02; Fig. 3G] with at least a pattern of increased lipid oxidation (Fig. S6). Moreover, the lipid/carbohydrate ratio tended to increase, suggesting a shift from carbohydrate to lipid oxidation [unpaired Student's *t*-test: *t*(33)=1.89, *P*=0.07; Fig. 3H].

### *Tanc2* disruption causes hepatocellular abnormalities and liver dysfunction

**Fig. 4.**
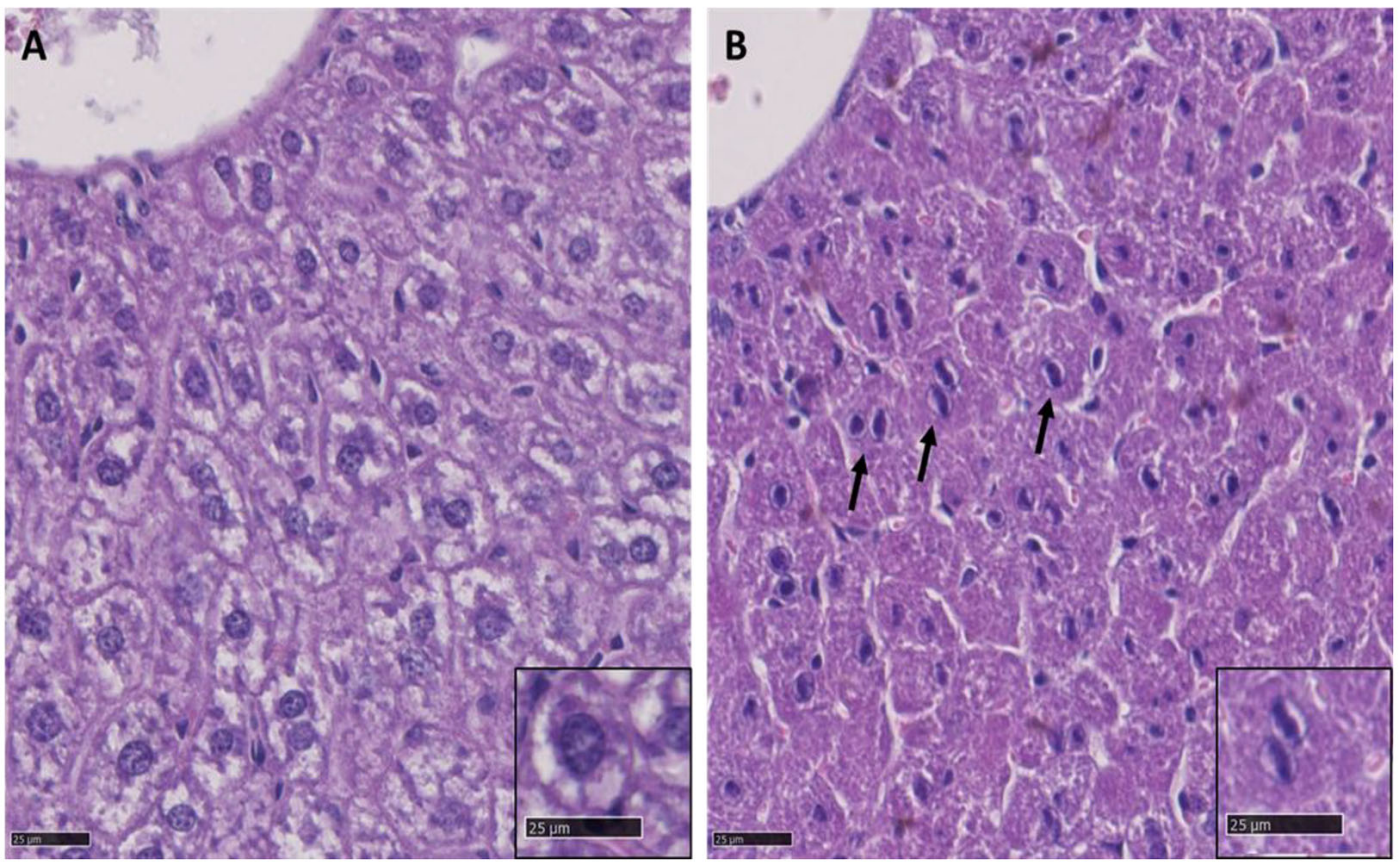
**Histopathological findings in the mouse liver at 16** **weeks of age.** (A,B) Representative liver sections stained with H&E, 800×magnification, from a control mouse (A) and from a *Tanc2*^−/−^ mouse (B). A shows the normal appearance of hepatocytes, with the round nucleus centrally located and cytoplasm containing glycogen. B shows, by comparison, in the liver of a *Tanc2*^−/−^ mouse, abnormal hepatocytes (arrows) with nuclear alterations consisting of linear chromatin with small lateral projections.

As already described, *Tanc2^−/−^* mice of both sexes were considerably lighter than their littermate controls and this was still evident at the end of the testing period (male +/+, 29.95±1.53 g; male −/−, 24.19±1.27 g; female +/+, 25.87±2.76 g; female −/−, 21.01±3.52 g; two-way ANOVA genotype effect: *P*<0.001) (16 weeks of age). Histological investigation of tissue from *Tanc2*^−/−^ mice at the age of 16 weeks (*n*=2 per sex) exhibited a liver phenotype consisting of focal rounded extensions of the hepatic lobes with nuclear alterations of hepatocytes with 75% penetrance (three of four) compared to controls (none of four; Fig. 4). A normal hepatocyte is polygonal with a granular dense cytoplasm and eosinophilic but may vary depending on nutritional status (glycogen accumulation in non-fasted adult mice). The nucleus is generally round with evenly dispersed chromatin and obvious nucleolus (Fig. 4A; Conrad et al., 2013). Binucleated hepatocytes are common. Nevertheless, *Tanc2* disruption caused nuclear alterations that comprised of linear chromatin with small lateral projections (Fig. 4B), which are associated with developmental anomalies previously described in the liver of rodents (Thoolen et al., 2010).

**Fig. 5.**
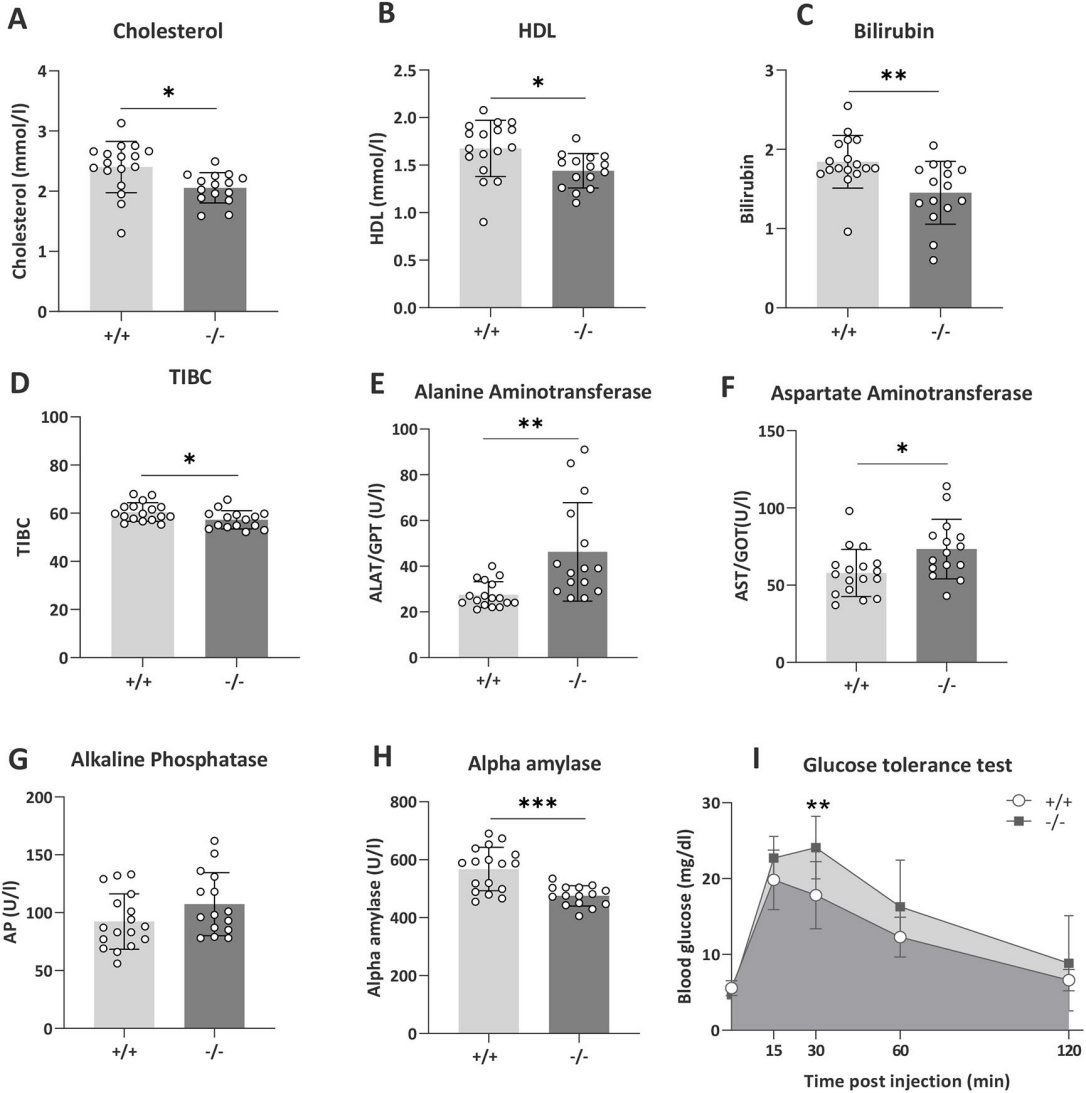
***Tanc2* disruption alters markers of liver damage.** (A,B) *Tanc2* disruption in −/− mice led to decreased circulating cholesterol (A) and high-density lipoprotein (HDL) levels (B), suggesting altered lipoprotein metabolism. (C,D) Bilirubin (C) and total iron binding capacity (TIBC; D) were decreased in −/− mice, indicating altered hepatocellular metabolism. (E,F) Alanine aminotransferase (E) and aspartate aminotransferase (F) levels were increased in −/− mice, signifying liver cell damage. (G,H) Alkaline phosphatase (G) tended to increase while alpha-amylase (H) decreased in −/− mice, implying altered hepatocellular function. (I) In the glucose tolerance test, the peak glucose level was increased in the mutant mice at 30 min post-glucose injection. *P<0.05, **P<0.01, ***P<0.001 +/+ versus −/− (unpaired Students's *t*-test or RM ANOVA with post-hoc Sidak's test). Data are mean±s.d., males and females pooled.

Liver function includes endocrine and exocrine activity, metabolism and detoxification. Thus, we determined how altered liver morphology and structural damage translated into altered markers of liver function and disease. *Tanc2* disruption was associated with a series of abnormalities emblematic of liver dysfunction (Fig. 5). In terms of lipoprotein levels, *Tanc2* disruption caused decreased circulating total cholesterol levels as well as decreased high-density lipoprotein (HDL) levels [unpaired Student's *t*-test: *t*(30)=2.75, *P*=0.01 (cholesterol); *t*(30)=2.66, *P*=0.01 (HDL); Fig. 5A,B; MP: 0005179 and 0000186, respectively]. Together, these changes indicate abnormal liver lipoprotein metabolism. Bilirubin is the product of haemoglobin catabolism, converted from unconjugated to conjugated forms in the liver. *Tanc2* disruption caused decreased bilirubin levels [unpaired Student's *t*-test: *t*(30)=3.04, *P*=0.005; MP: 0005635] and also decreased total plasma iron-binding capacity [TIBC; unpaired Student's *t*-test: *t*(30)=2.36, *P*=0.02], a surrogate marker of the levels of plasma transferrin, a liver-derived protein, further hinting towards altered hepatocellular metabolism (Fig. 5C,D). Plasma activities of several enzymes, with significant liver-derived contribution to total activity measured, were altered in mice with *Tanc2* disruption: alanine and aspartate aminotransferase (AT) levels both increased, inferring liver cell damage [unpaired Student's *t*-test: *t*(30)=3.46, *P*=0.002 (alanine AT); *t*(30)=2.54, *P*=0.02 (aspartate AT); Fig. 5E,F]. Furthermore, alkaline phosphatase (AP) levels tended to increase [unpaired Student's *t*-test: *t*(30)=1.66, *P*=0.11; MP: 0002968], whereas alpha amylase [unpaired Student's *t*-test: *t*(30)=4.36, *P*=0.0001; MP: 0008806] levels were decreased. Besides pancreas and salivary glands, the liver is a major source of plasma alpha amylase activity in rodents (MacKenzie and Messer, 1976; Sierra et al., 1986), and AP is one of the diagnostic markers of cholestasis in mice (Hochrath et al., 2013) and humans (Gunaydin and Bozkurter Cil, 2018) (Fig. 5G,H).

*Tanc2* disruption was associated with higher peak blood glucose levels 30 min after injection in the glucose tolerance test (GTT) (Fig. 5I), although the differences were not significant at the other time points post-injection [RM ANOVA: time×genotype interaction effect *F*(4, 120)=6.30, *P*=0.0001, post-hoc Sidak's multiple comparisons: 30 min, *P*=0.0013]. Overall, this indicates higher peak glucose levels and delayed glucose clearance consequent to *Tanc2* interference, suggesting impaired glucose tolerance (MP: 0005293)*.*

### Dimensionality reduction of psychiatric and systemic phenotypes with *Tanc2* disruption

Given the multifaceted pathology associated with *Tanc2* disruption, we wanted to evince a visible phenotypic cluster profile, distilling the most salient features into potential multimodal biomarkers of central nervous system disturbance in NDDs. We therefore implemented an exploratory principle component analysis (PCA) for unsupervised pattern recognition and variable dimensionality reduction to reveal covariance structure and phenotypic combinations that best typify *Tanc2* disruption pathology.

**Fig. 6.**
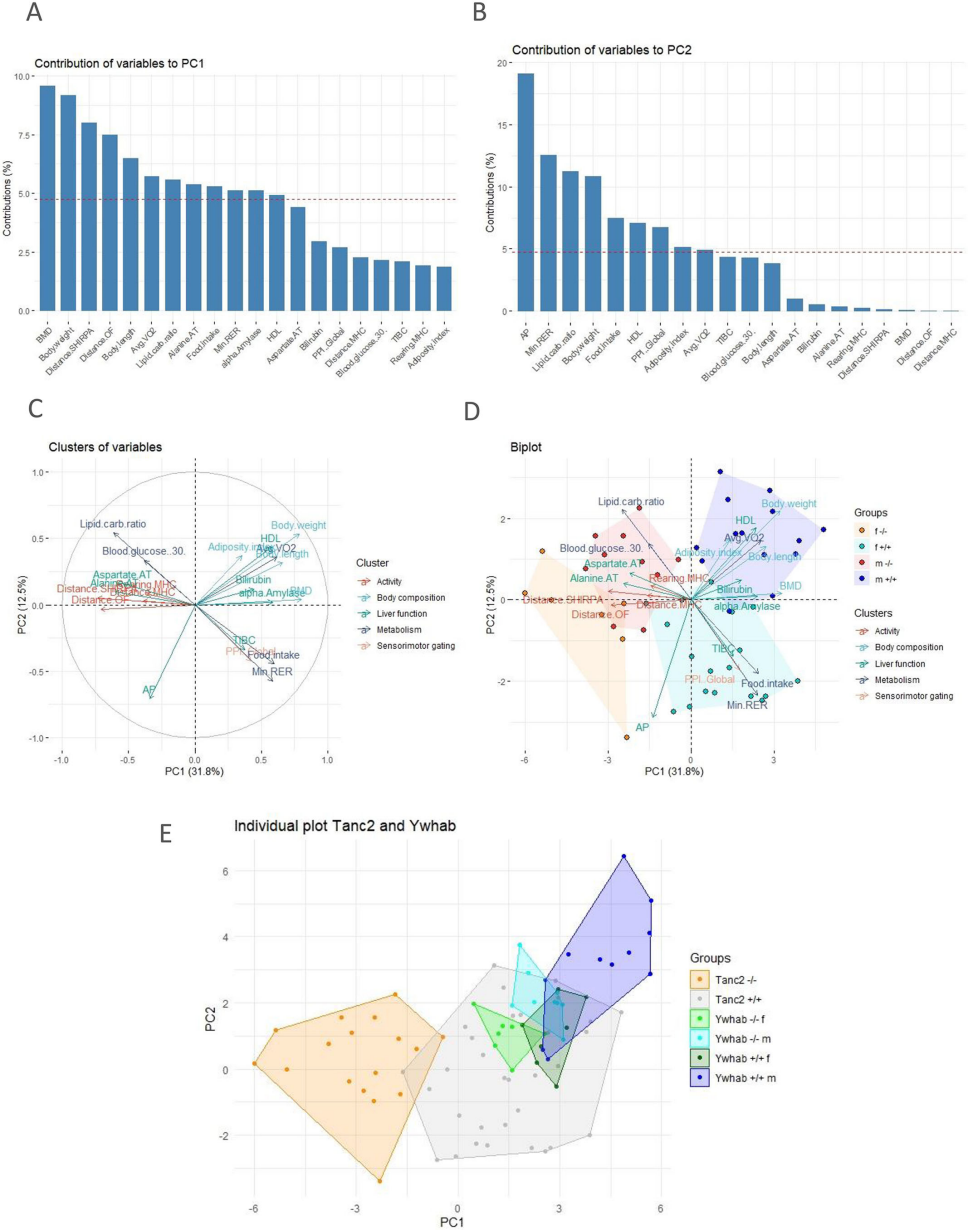
**Dimensionality reduction of psychiatric and systemic variable effects of *Tanc2* disruption.** We performed a principal component analysis (PCA) to reduce the dimensionality of the variables related to *Tanc2* disruption. (A,B) The contribution of variables to principal component (PC) 1 (A) and 2 (B). (C) Correlation circle showing clustering of variables in PC1 and 2. (D) Biplot depicting the variables and individual PC scores for the two genotypes, control (+/+) and *Tanc2*-disrupted mutants (−/−). Males and females were separated for PCA. (E) Individual plot of *Ywhab*^−/−^ mice and control PC scores with *Tanc2*^−/−^ mice PC scores and their respective controls. The coloured clouds denote animals from the same experimental group. AP, alkaline phosphatase; AT, aminotransferase; BMD, bone mineral density; carb, carbohydrate; HDL, high-density lipoprotein; MHC, metabolic home cage; OF, open field; PPI, prepulse inhibition; Min RER, minimum respiratory exchange ratio; TIBC, total iron-binding capacity.

In practice, a PCA uses linear weighted mixtures of measured phenotypes to unearth the primary orthogonal (uncorrelated) components or factors that explain the total variance. Two major principal components (PCs) explained ∼45% of the phenotypic variance. Analysis of the loadings (weights of the original variables) indicated that PC1, which accounted for 31.8% of the variance, loaded most highly (>5% contribution) for the following: BMD, body weight, distance SHIRPA, distance OF, body length, average VO_2_, lipid/carbohydrate ratio, alanine AT, food intake, minimum RER, alpha amylase and HDL (Fig. 6A). AP, minimum RER, lipid/carbohydrate ratio, body weight, food intake, HDL, PPI global, adiposity index and average VO_2_ loaded highly on PC2, which accounted for 12.5% of the variance (Fig. 6B).

To scrutinise further the two components, we generated a correlation circle between the variables and the components (Fig. 6C). The variables can be clustered into different groups, further depicting their influence on the PCs. PC1 is characterised by four distinct clusters: (1) activity (in both novel and homecage environments); (2) body composition (BMD, body weight, body length); (3), metabolism (average VO^2^, lipid/carbohydrate ratio, food intake and minimum RER); and (4) liver function (alanine AT, alpha amylase and HDL). Four clusters appear in PC2: (1) liver function with AP and HDL; (2) metabolism (minimum RER, lipid/carbohydrate ratio, food intake and average VO_2_); (3) body composition (body weight and adiposity index); and (4) sensorimotor gating (PPI global).

Using loadings from the first two PCs, we calculated two composite PC scores, denoting the coordinates of each animal in the space given by the PCs, where the scores represented a designated phenotype (PC1 and PC2). This allows the identification of animal groups showing similar (close to each other in the graph) or different (far away from each other) phenotypes. The clustering of the individual scores indicates that there is a common multivariate combination of phenotypes underlying this clustering pattern. It is therefore apparent that PC1 separates the control (+/+) from the *Tanc2*-disrupted (−/−) groups (Fig. 6D). The greater the % contribution of a phenotype loading to a PC, the more that particular phenotype accounts for the differences in animals with high scores within that PC. Thus, we could distinguish best the two genotypes by the combination of hyperactivity with strongly correlated altered liver damage markers as well as growth failure.

To determine whether PC1 could also distinguish mice mutant for the Hippo pathway signalling gene *Ywhab*, we calculated PC scores for −/− mutant and control mice that ran through the same phenotyping pipeline, and data are available at https://www.mousephenotype.org/data/search?term=ywhab&type=gene. As seen in Fig. 6E, where we plotted the PC scores for the *Ywhab* mice alongside those for the *Tanc2* mice, PC1 was able to separate the *Ywhab*^−/−^ mice from controls.

## DISCUSSION

NDDs, while predominated by neuropsychiatric manifestations, are frequently associated with medical comorbidity of potential genetic origin. Using a homozygous viable *Tanc2* disruption model, we highlight the pleiotropic effects of TANC2 mutation on somatic disease risk associated with NDD. As well as precipitating NDD-like behavioural features in mice, *Tanc2* disruption caused growth failure, a shift in adiposity and liver damage with associated abnormal liver function. Such aberrations will have long-term implications for individual health, emphasising the importance of somatic disease-risk portent screening with autosomal-recessive *TANC2* mutation. The IMPC database is thus a valuable tool to forewarn of and evaluate the medical comorbidity associated with pleiotropic disease-causing NDD genes.

The conservation of phenotypes across vertebrates infers conservation of gene function. Thus, for rare high-risk disease-causing genes, a preclinical disruption model system is of immense value to understand gene function and disease pathogenesis, and to probe the underlying mechanisms involved. This is especially true given the difficulty in recruiting sufficient patients with such rare mutations from whom to glean information. Furthermore, the use of systematic human and mouse ontologies in databases permits semantic interoperability in digital medicine and novel integrative and machine learning approaches to understand molecular disease underpinnings (Schofield et al., 2010). Whereas a previously generated homozygous *Tanc2* knockout mouse model was embryonic lethal (Han et al., 2010), the homozygous *Tanc2*-disrupted mice described here were viable. The variation in viability likely relates to the use of two different targeting strategies and genetic strains (CRISPR/Cas on C57BL/6N versus Genetrap on unknown background). It is therefore consistent with the already known contextual nature of essential gene functions, at least in human cells, and some differences between CRISPR and Genetrap approaches in identifying cell-essential genes (Wang et al., 2015; Bertomeu et al., 2018). Heretofore undefined genetic modifiers and epigenetic variation between different background strains may alter how the two mutagenesis approaches influence the essentialome. This *Tanc2* disruption model therefore has translational value for autosomal-recessive variant cases. Evidence indicates that TANC2 is dosage sensitive (Wessel et al., 2017), with the potential for similar albeit milder effects expected for specific heterozygous *TANC2* variants. The model thus provides a platform to elucidate both the developmental and adult-derived effects of biallelic *Tanc2* disruption.

Regarding the phenotypes, the clear hyperactivity in these young adult mutant mice is consonant with the hyperactivity exhibited by at least a subset of patients harbouring autosomal-dominant *TANC2* mutations (Wessel et al., 2017; Guo et al., 2019). The affected probands ranged in age from children (>5 years) to young adults (<31 years), but more analysis is needed to determine whether *Tanc2* disruption increases activity in pre-weanling mice. Hyperactivity was also associated previously with loss of other post-synaptic scaffold proteins such as ProSAP1/SHANK2 (Schmeisser et al., 2012). Within the post-synaptic density, scaffolding proteins connect surface receptors with intracellular effectors and thereby influence synaptic plasticity by affecting receptor distribution (Iasevoli et al., 2013). The marked hyperactivity consequent to *Tanc2* disruption is therefore likely due to impaired synaptic structure and function and thus the culmination of altered glutamatergic neurotransmission and excitation/inhibition imbalance (Schmeisser et al., 2012). A recent study supports this assertion, whereby *Tanc2* haploinsufficiency also altered NDD-relevant behaviours in mice. These effects included hyperactivity and suppressed long-term potentiation (LTP) at Schaffer collateral-CA1 pyramidal cell synapses, but largely normal social behaviour in young adult mice (Kim et al., 2021). Treatment of *Tanc2* haploinsufficient mice with rapamycin during a pre-weaning critical axonal and dendritic developmental time window (postnatal days 5-14) successfully rescued the hyperactivity and LTP phenotypes. *Tanc2* disruption can therefore untether mTOR pathway activity, impeding normal synaptic development with behavioural consequences relevant to NDDs. Impaired PPI, an index of sensorimotor gating, is an endophenotype of NDDs including schizophrenia that can be due also to altered excitatory neurotransmission with adult-based or developmental origins (DiLalla et al., 2017; Perry et al., 2007; Geyer et al., 2001). Notably, that *Tanc2* disruption impairs PPI at less-salient PP intensities [2, 4 (trend) and 8 dB above background noise] and not at the higher intensity (at 16 dB above background) suggests that a specific microcircuit is affected rather than a global obstruction of sensorimotor gating function. Further analysis is needed to detail this abnormality; however, differences in PPI at lower versus higher PP intensities were associated with alterations in mesolimbic dopaminergic activity that may be altered in these mice (Yee et al., 2005).

TANC2 is highly expressed during brain development and interacts with proteins from important developmental signalling pathways such as Wnt (PRICKLE1/2) and Hippo (ZYX, YWHAB, LATS2), as well as with proteins involved in cilium assembly (CBY1, CEP120) and trafficking (INPP5E) (Gasparini et al., 2017). Both Wnt and Hippo pathway proteins can regulate, among other neurodevelopmental processes, dendritic arborisation and synapse formation (Budnik and Salinas, 2011; Sahu and Mondal, 2021). Thus, TANC2 can influence synaptic function through interaction with these pathways leading to NDDs. For example, PRICKLE2, a post-synaptic non-canonical Wnt signalling protein associated with ASD, interacts with both TANC2 and PSD95. Disruption of *Prickle2* in mouse hippocampal neurons caused decreased dendritic branching and synapse number (Sowers et al., 2013). The Hippo pathway-related protein PPP1CC, predicted to interact with TANC2, is part of the protein phosphatase 1 (PP1) subfamily. PP1 protein, as well as regulating cell division, influences long-term synaptic plasticity and brain function (Foley et al., 2021). Further analysis of this model should yield insights into the precise developmental origins of the underlying alterations involved.

As well as behavioural manifestations of *Tanc2* disruption, there were systemic alterations. The apparent constitutional growth failure (lower body weight) and short stature (decreased body length) in young adults were associated with a slew of protracted and correlated dysmorphological and metabolic alterations. These included decreased BMC as well as decreased oxygen consumption and metabolic rate. Growth failure is frequently associated with NDDs such as Rett syndrome (Tarquinio et al., 2012). Nevertheless, the rate of body weight gain was normal, albeit on a lower elevation, implying early maldevelopmental origin of growth failure sustained into adulthood. The shift in adiposity index towards less fat mass in favour of lean mass is consistent with the role of TANC2 in muscular adiposity in fish (Zheng et al., 2016), pigs (Liu et al., 2009) and cattle (Ishii et al., 2013). *Tanc2* was upregulated in pig longissimus muscle in those with higher fat (Liu et al., 2009). *Tanc2* disruption therefore influences body composition, adiposity and body size.

There was also evidence of liver dysfunction with *Tanc2* disruption. Altered hepatocyte development precipitated abnormal tissue morphology and circulating liver function markers (Thoolen et al., 2010). Although the mechanism requires investigation, predicted TANC2 interaction proteins reveal clues. This includes PRICKLE1 regulating canonical Wnt/beta-catenin signalling and modulating developmental planar cell polarity (polarisation of cells within an epithelial sheet) and ciliogenesis (Gibbs et al., 2016). *Prickle1* underexpression, with consequent abnormal Wnt signalling, causes hepatocellular carcinoma (Chan et al., 2006). Hepatoblast proliferation within liver growth zones necessitates also beta-catenin signalling (Ober and Lemaigre, 2018). Together, this suggests that *Prickle1* and Wnt signalling irregularity may trigger some *Tanc2* disruption-related defects. Additionally, the Hippo pathway regulates liver size, leading to liver cancer when dysfunctional (Ober and Lemaigre, 2018). TANC2 interacts with several Hippo pathway proteins including YWHAB and PPP1CC. IMPC data indicate that interference of these proteins phenocopies the altered indices of lipoprotein metabolism seen with TANC2 disruption, illustrating their importance for liver function (www.mousephenotype.org, search terms ‘*Ywhab*’ or ‘*Ppp1cc*’ for phenotyping results). There are no reports of hepatic impairment in patients with autosomal-dominant *TANC2* mutation thus far (Guo et al., 2019), although at least one patient in this study exhibited obesity with fatty liver. Nevertheless, gone undetected, abnormal liver function can potentially increase long-term somatic disease risk. For instance, decreased HDLs augment cardiovascular disease susceptibility through arteriosclerosis (Kuai et al., 2016). Furthermore, the delayed glucose clearance likely stems from impaired hepatic glucose metabolism, spurring diabetes (Petersen et al., 2017). Additionally, decreased antioxidant bilirubin can lead to cardiovascular disease and stroke, with secondary effects of diabetes and dyslipidaemia (Chung et al., 2019; Takei et al., 2019; Maruhashi et al., 2019). It is also plausible to suggest a feedback relationship between liver disease and central nervous system alterations (Jones and Weissenborn, 1997; Jayanti et al., 2020), with mechanisms similar to those observed in hepatocerebral degeneration (Shin and Park, 2017). Therefore, our results suggest that, besides direct effects on neurological function, TANC2 disruption may increase susceptibility to systemic disease.

There is an unmet need to understand fully the pathoaetiology of NDDs and identify translational biomarkers for early NDD diagnosis and novel treatments. Effective biomarkers will index the abnormal pathways underlying the behavioural outcomes scrutinised in clinical trials and be relevant for treatment response. With this interdisciplinary approach, we observed that biallelic *Tanc2* disruption induced a distinct multidimensional profile of psychiatric and systemic effects. The PC (PC1) that most clearly embodied *Tanc2* disruption encompassed three marked phenotypic clusters likely of developmental origin. Therein, the covarying hyperactivity with liver dysfunction indices, for example, may indicate a causative relationship. Alternatively, these changes may covary due to similar developmental pathway interference as alluded to previously, e.g. Hippo or Wnt, a prospect supported by the ability of PC1 to distinguish mice mutant also for the Hippo gene *Ywhab*. If the latter, then such a multi-systemic symptom cluster could signify *TANC2* disruption in NDD patients for confirmation with molecular profiling. Circulating indices of liver dysfunction can thus serve as a novel and easily accessible biomarker in this case.

In summary, we here identified a homozygous viable disrupted *Tanc2* model system that allowed us to assess the function of this NDD-causing gene beyond embryogenesis into adulthood. The evident behavioural alterations akin to human NDD patients highlight the translational value of this preclinical model. In addition, we determined the pleiotropic effects of the *Tanc2* gene beyond the brain, effects such as hepatic dysfunction that can predispose to somatic disease development long term. In the interests of improved patient care, this outcome emphasises the importance of holistic multi-systemic approaches to NDD diagnosis and treatment in cases with high-risk *TANC2* gene, particularly biallelic, mutation. Future analyses will elucidate the details of how TANC2, and potentially other convergent post-synaptic scaffold proteins within the same pathway and protein class, underlie the shared genetic architecture of NDDs and the complex traits related to medical comorbidity. Moreover, the large-scale IMPC preclinical database is a valuable tool to identify not just the shared genetic risk of NDD psychiatric and somatic manifestations but also the overlapping abnormalities with biomarker potential.

## MATERIALS AND METHODS

### Animals

The *Tanc2^-em1/CRISPR/Cas^* mouse model was generated using the IMPC targeting strategy with CRISPR/Cas technology (https://www.mousephenotype.org/understand/the-data/allele-design/) at Helmholtz Zentrum München as follows. Single-guide RNAs (sgRNAs) were selected [CRISPR Design Tool: http://crispor.tefor.net/ (Haeussler et al., 2016)] and a 4-guide (4G) approach was used to generate *Tanc2* exon 5 deletion alleles (Gene ID: 26115; see Fig. S1A,B for guide and rest protein details). The 4G strategy was used to improve the cutting efficacy and is a general method in the IMPC consortium (https://www.mousephenotype.org/understand/start-using-the-impc/allele-design/). All the guides have no off targets in protein-coding regions of the mouse genome (Fig. S2). SgRNAs were *in vitro* transcribed [EnGen Kit, E3322S, New England Biolabs (NEB)] and primers generated (NEB Tool: http://nebiocalculator.neb.com/#!/sgrna, Metabion). Following *in vitro* transcription, RNA was purified (RNA Clean & Concentrator™-25, R1017, Zymo). The deletion allele ribonucleoprotein (RNP) electroporation mix consisted of Cas9 protein (200 ng/µl) and sgRNA (50 ng/µl each; 200 ng/µl in total) in a final volume of 7.5 μl water (RNAse free). Immediately before electroporation, an equal volume of 2× OptiMEM (31985062, Thermo Fisher Scientific) was added.

Thirty-two-day-old C57BL/6NCrl female mice were injected with pregnant mare serum gonadotropin (5 IU/mouse) followed 48 h later with human chorionic gonadotropin (5 IU/mouse). The females were then mated to C57BL/6NCrl males, and fertilised oocytes were collected [0.5 days post-coitum (dpc)]. Zygotes were washed once in OptiMEM (T1788, Sigma-Aldrich). Then, 5 µl of the electroporation solution (RNP Complex in OptiMEM) was filled into the gap between the electrodes of a CUY501P1-1.5 electrode (1 mm gap) from NEPA Gen and electroporated with a standardised protocol. After electroporation, the embryos were rinsed through HTF medium and cultivated overnight. Two-cell embryos were then transferred into pseudopregnant (0.5 dpc) CD-1 females the next day. Mice were kept in individually ventilated cages with water and standard mouse chow available *ad libitum* according to the directive 2010/63/EU. The care and use of animals used in this study was approved by and complied with the rules of the ethical committee of the district government of Upper Bavaria (Regierung von Oberbayern), Germany, and conducted according to rules and policies outlined by the ethical committee of Helmholtz Zentrum München.

Genomic DNA was extracted from tissue samples collected from mice during ear labelling at weaning (Wizard Kit, A1120, Promega), and PCR reaction was performed with *Tanc2*-specific primers (Tanc2-1 forward, 5′-tcattagattctgtgtagtgatct-3′; Tanc2-1 reverse, 5′-aagcatactatttaatactttagca-3′). DNA was amplified by PCR using LongAMP polymerase (M0323L, NEB). The two primers bind outside the two sgRNA sites to PCR amplify deletion products. All PCR products were visualised using the Gene Tools System from SynGene. The PCR products were directly sent for sequencing to the Eurofins Genomics Company. The genotyping results are shown in Fig. S1D. RNA quality control analysis using RT-PCR of heterozygous splenic tissue (Fig. S1E) revealed that the mutation (deletion exon 5) was successful and the full-length protein is null. There were two small transcript fragments remaining that were unlikely translated (Fig. S1F) because of incomplete 5′ coding sequence. Founders born from microinjection or electroporation experiments that carried the desired allele based on genotyping results were pair mated to C57BL/6NCrl mice. Born N1 pups were screened with the same genotyping assay as used to identify founders. Heterozygous×heterozygous matings were established to generate sufficient −/− mice with littermate +/+ controls for experimental analysis. The *Tanc2*^−/−^ mice can be ordered through the IMPC website (www.mousephenotype.org/data/genes/MGI:2444121#order).

### Mice phenotyping and body weight analysis

From the age of 8-16 weeks, the *Tanc2^-em1/CRISPR/Cas^* mice were systematically phenotyped in the German Mouse Clinic, as described previously (Fuchs et al., 2018) and in accordance with the standardised phenotyping pipeline of the IMPC (IMPReSS: https://www.mousephenotype.org/impress/index). The testing details described here are for those assays in which *Tanc2*-pertinent alterations were detected. Homozygous mutant (−/−) and wild-type littermate controls (+/+) were compared, and the number of animals per group and age of testing for the different assays are shown in Table S1. For the analysis of body weight throughout the phenotyping period, a linear mixed-effects model was applied with the fixed effects genotype, age and the interaction of these two. All data are available on the IMPC database (https://www.mousephenotype.org/data/genes/MGI:2444121) and are free to download and analyse.

### OF

The 20-min OF test was carried out at 8 weeks of age using the ActiMot system (TSE, Germany) as described previously (Garrett et al., 2012). The arena was made of transparent and infrared light-permeable acrylic with a smooth floor (internal measurements, 45.5×45.5×39.5 cm; illumination, 150 lux corners, 200 lux middle).

### SHIRPA

The SHIRPA test evaluated pronounced physical characteristics, behaviours and morphological aberrations at 9 weeks of age. Defined rating scales (as expected/not as expected, present/absent, reduced/normal/increased) were used and the squares-crossed number (first 30 s after transfer) indexed locomotor activity.

### PPI of acoustic startle

Acoustic startle response (ASR) and PPI were examined at 10 weeks of age with modification to the previously described protocol (Heermann et al., 2019); further details can be found at https://www.mousephenotype.org/impress/ProcedureInfo?action=list&procID=746&pipeID=14. Briefly, the Med Associates Inc. (St Albans, VT, USA) startle equipment was used with background noise [no stimulus (NS)] set to 65 dB. Basal startle response (S; startle pulse of 110 dB/40 ms white noise) and % PPI [to four different PP intensities (67, 69, 73, 81 dB; 2, 4, 8 and 16 dB above background, respectively), 50-ms interval between S and PP] were determined.

### Indirect calorimetry in MHCs

At the age of 11 weeks, MHC locomotor activity (distance travelled) and exploration (rearing), gas exchange (oxygen consumption and carbon dioxide production, VCO_2_/VO_2_), energy expenditure (heat production, kJ/h/animal), food intake and substrate utilisation of single-caged mice was measured by indirect calorimetry in metabolic homecages (TSE, Germany) (detailed protocol: https://www.mousephenotype.org/impress/ProcedureInfo?action=list&procID=855&pipeID=14). The measurement commenced 5 h before lights off and finished 4 h after lights on the next morning (21 h in total).

### Body composition (DEXA lean/fat)

The BMC, BMD and body composition were assessed using a dual energy X-ray absorptiometry (DEXA; Faxitron Bioptics LLC, Tucson, AZ, USA) analyser at the age of 14 weeks. The body length was also measured along a ruler. The procedure was performed according to the open-access IMPC testing standard operating procedure (https://www.mousephenotype.org/impress/ProcedureInfo?action=list&procID=554&pipeID=14). In brief, mice were anaesthetised and placed in the DEXA analyser. A snout and measure scan were performed, and regions of interest were defined. The standard analysis focuses on the whole body excluding the head. The mouse rests on a heating mat at 37°C until conscious again.

### GTT

Glucose metabolism disturbance was determined using the GTT at the age of 13 weeks. Glucose was administered intraperitoneally (2 g/kg) after a 16-h food withdrawal, and glucose levels were measured 15, 30, 60 and 120 min later. Basal fasting blood glucose level was analysed with an Accu-Chek Aviva Connect glucose analyser (Roche/Mannheim).

### Blood collection and clinical chemistry

Final blood samples were collected under isoflurane anaesthesia by retrobulbar puncture in Li-heparin-coated tubes and stored on ice until centrifugation (4500 ***g***, 10 min) and separation of plasma aliquots for further analyses. The clinical chemistry analyses of circulating biochemical parameters in *ad libitum*-fed mouse blood was performed using a clinical chemistry analyser (Beckman Coulter AU 480 autoanalyser, Krefeld, Germany) at the age of 16 weeks. A broad set of parameters was measured, including enzyme activities as well as plasma concentrations of specific substrates and electrolytes (Rathkolb et al., 2013).

### Pathological examination

For pathological analyses at 16 weeks of age, Hematoxylin and Eosin (H&E) staining was performed on formalin-fixed paraffin-embedded sections (4 µm) from 28 organs. Two independent pathologists analysed the slides according to standardised protocols as previously described (Fuchs et al., 2018).

### Statistics

The data presented here were garnered using the standardised large-scale phenotyping pipeline of the IMPC (see https://www.mousephenotype.org/impress/index) in which a series of different assays was implemented. Such an approach necessarily introduces caveats related to false-positive detection and data interpretation as outlined previously (Maier et al., 2017). It should thus be borne in mind that a correction for multiple testing has not been performed. Data were analysed using two-way ANOVA with post-hoc Tukey's to test genotype×sex interaction effects. When no clearly significant interaction effects were detected with narrow 95% confidence intervals, the data for males and females were collapsed for specific parameters and the pooled data compared using unpaired Student's *t*-test. For indirect calorimetry analysis over 21 h, including distance and rearing, the light and dark phases were analysed separately using RM ANOVA (with post-hoc Sidak's test) with time and genotype as independent variables. Linear regression analysis was used to determine how body weight was a predictor of metabolic and dysmorphological measures. Data were statistically analysed using Prism version 8 for Windows (GraphPad Software, La Jolla, CA, USA; www.graphpad.com). For all tests, a *P*-value<0.05 was the level of significance and data are presented as mean±s.d. For the PCA, missing data in the dataset were imputed by the ‘missMDA’ package in R. The analysis was performed using SPSS for the whole dataset. Data were standardised via Z-transformation. Sampling adequacy was confirmed by the Kaiser–Meyer–Olkin criteria (KMO=0.7) and the Bartlett test (χ²=597.4, d.f.=210, *P*<0.001). Subsequent analysis and visualisation was done in R with the ‘FactoMineR’ and ‘factorextra’ package.

## Supplementary Material

Supplementary information
