# Supplementary material for: Post-synaptic scaffold protein TANC2 in psychiatric and somatic disease risk
Source: Dis Model Mech. 2022 Mar 4;15(3):dmm049205. doi: 10.1242/dmm.049205 (PMC8906171; doi:10.1242/dmm.049205)
Supplement: Supplementary information [file dmm-15-049205-s1.pdf]

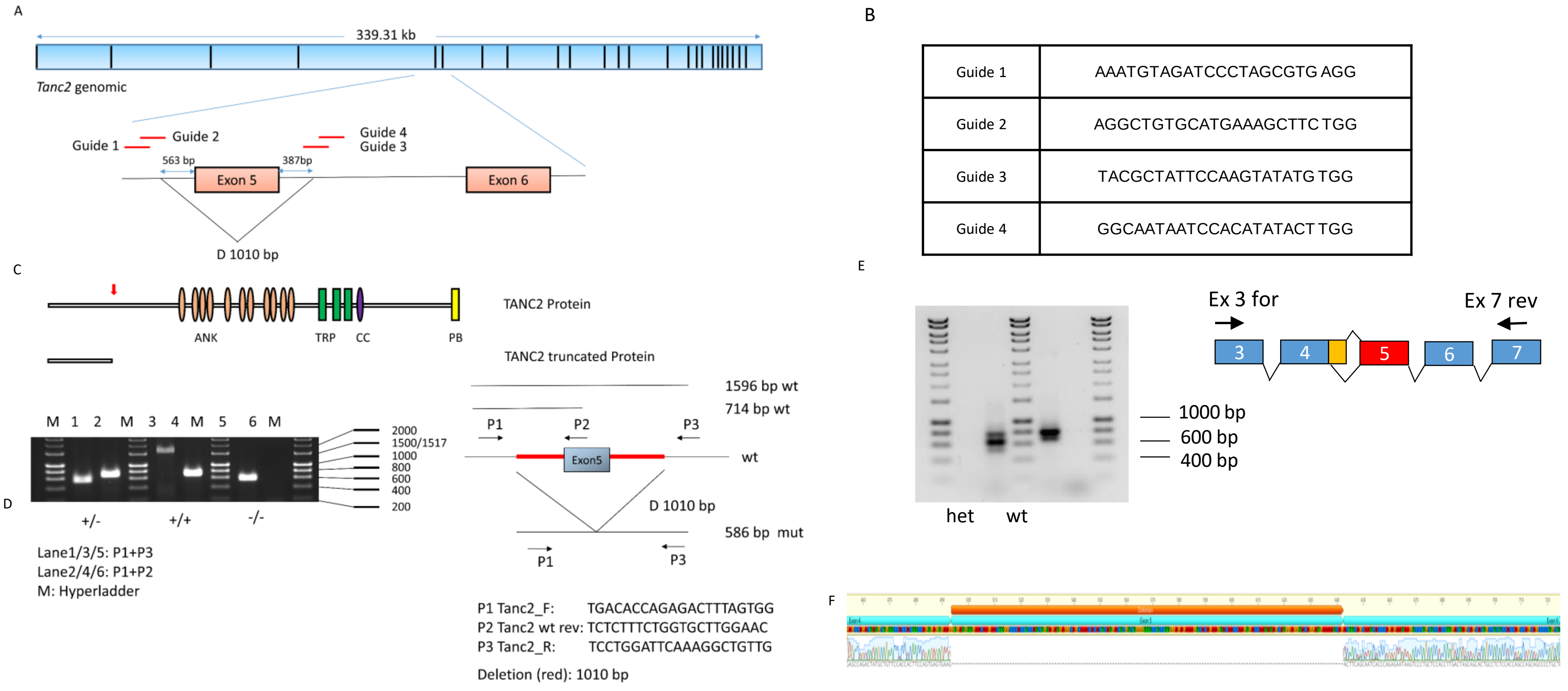

Fig. S1. Generation of TANC2-deficient mice. Schematic diagrams showing the locations of the guides used to delete exon 5 (**A, B**) and the site of protein truncation (**red arrow**) (**C**) Information about Guide coordinates and mutation are also available at www.gentar.org. The genotyping strategy is shown (**D**) for detection of *Tanc2* mutants by PCR. Primer pair P1-P3 can be used for the detection of wildtype as well as mutant. RNA quality control (QC) result (E) that was carried out on a spleen from a *Tanc2* +/- mouse. RT-PCR analysis and sequencing of the isolated fragments from wt and heterozygous mice show two bands for wt of 695 bp and 810 bp (not annotated splice variant with 111bp intron sequence (orange) 3‘ to exon 4) and for the heterozygous animals three bands of 810 bp (wt extended exon 4) 661 bp (deletion exon 5 in red with exon 4 entension) and 546 bp (deletion exon 5; Fig. **F**). Deletion of exon 5 in the 4th exon extension also shows a frameshift.

Disease Models & Mechanisms • Supplementary information

Off-Target

Position/ Strand	Guide Sequence + *PAM* + Restriction Enzymes ☐ Only G- ☐ Only GG- ☐ Only A-	MIT Specificity Score	CFD Spec. score	Predicted Efficiency (Show all scores): Doench '16	Predicted Efficiency: Mor.-Mateos	Outcome: Out-of-Frame	Outcome: Lindel	Off-targets for 0-1-2-3-4 mismatches + next to PAM	Genome Browser links to matches sorted by CFD off-target score (No exons. ☑ CM004225.1 only)
63 / fw	AAATGTAGATCCCTAGCGTG *AGG*<br>**Cloning / PCR primers**	90	94	63	68	56	78	0 - 0 - 0 - 4 - 51<br>0 - 0 - 0 - 0 - 1<br>55 off-targets	4:CM004225.1 90.33 Mbp<br>**get primers**
83 / fw	AGGCTGTGCATGAAAGCTTC *TGG*<br>⚠ Inefficient<br>Enzymes: *AluBI, HindIII, Hpy188III*<br>**Cloning / PCR primers**	70	86	38	47	84	75	0 - 0 - 1 - 19 - 142<br>0 - 0 - 0 - 2 - 4<br>162 off-targets	4:CM004225.1 78.18 Mbp<br>4:CM004225.1 88.99 Mbp<br>4:CM004225.1 128.81 Mbp<br>3:CM004225.1 83.29 Mbp<br>**Off-target primers**
83 / fw	TACGCTATTCCAAGTATATG *TGG*<br>**Cloning / PCR primers**	86	94	55	14	67	84	0 - 0 - 1 - 8 - 52<br>0 - 0 - 0 - 1 - 4<br>61 off-targets	4:CM004225.1 34.11 Mbp<br>4:CM004225.1 2.54 Mbp<br>4:CM004225.1 51.03 Mbp<br>**Off-target primers**
72 / rev	GGCAATAATCCACATATACT *TGG*<br>**Cloning / PCR primers**	80	91	52	4	60	67	0 - 0 - 0 - 5 - 100<br>0 - 0 - 0 - 0 - 1<br>105 off-targets	4:CM004225.1 68.03 Mbp<br>4:CM004225.1 37.76 Mbp<br>4:CM004225.1 85.70 Mbp<br>4:CM004225.1 80.59 Mbp<br>**Off-target primers**

```
guide:      AGGCTGTGCATGAAAGCTTC TGG
off-target: AGGCTGTGCTTGAGATCTTC CGG
                     *    * *
```

CFD Off-target score: 0.000000
MIT Off-target score: 0.06
Position: CM004225.1:83285491-83285513:-
Distance from target: 29.110 Mbp

Fig. S2. *Tanc2* disruption using CRISPR/Cas9 genome editing – potential off-target identification. All the guides have no off targets in protein coding regions in the mouse genome

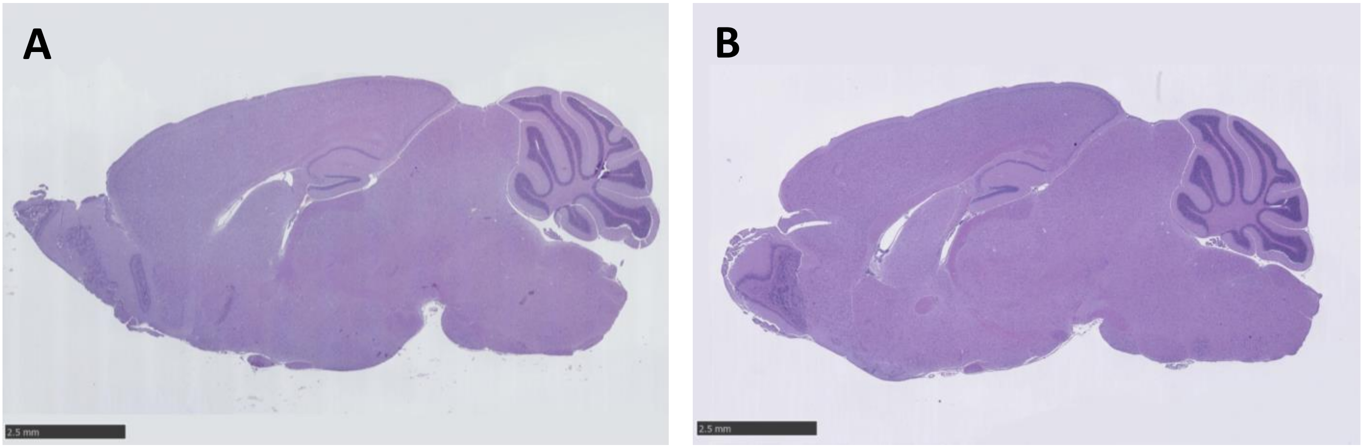

Fig. S3. Representative photomicrographs of sagittal brain sections stained with H&E, 12.5x magnification, from a control +/+ mouse (A) and from a *Tanc2* -/- mouse (B) at 16 weeks of age showing no obvious size differences between the genotypes, scale bars = 2.5mm

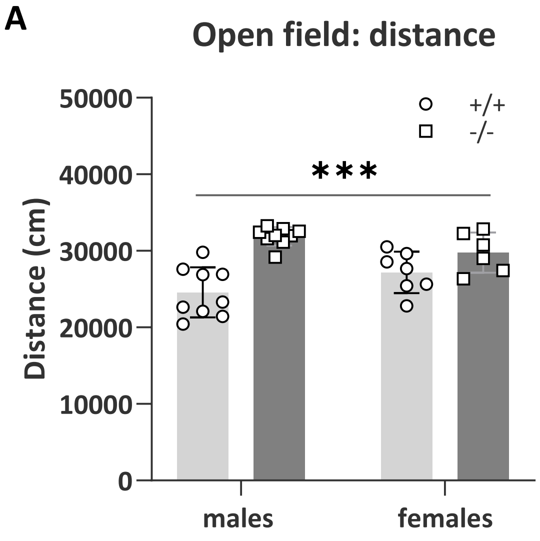

B

2way ANOVA
ANOVA results

ANOVA table	SS (Type III)	DF	MS	F (DFn, DFd)	P value
Interaction	41904231	1	41904231	F (1, 27) = 6.424	P=0.0174
sex	439232	1	439232	F (1, 27) = 0.06734	P=0.7972
genotype	185075960	1	185075960	F (1, 27) = 28.37	P<0.0001
Residual	176114755	27	6522769		
**Difference between column means**					
Predicted (LS) mean of +/+	25883				
Predicted (LS) mean of -/-	30843				
Difference between predicted means	-4960				
SE of difference	931.2				
95% CI of difference	-6871 to -3050				
**Difference between row means**					
Predicted (LS) mean of males	28242				
Predicted (LS) mean of females	28483				
Difference between predicted means	-241.6				
SE of difference	931.2				
95% CI of difference	-2152 to 1669				
**Interaction CI**					
Mean diff, A1 - B1	-7320				
Mean diff, A2 - B2	-2600				
(A1 -B1) - (A2 - B2)	-4720				
95% CI of difference	-8542 to -899.1				
(B1 - A1) - (B2 - A2)	4720				
95% CI of difference	899.1 to 8542				

Genotype effect

Interaction effect

Fig. S4. Distance travelled in the open field. The distance travelled tended to be higher in the males during the 20 minute open field test, ***p<0.001 genotype effect (**A**) however the 95% confidence interval (CI) was significantly more narrow for the main genotype effect vs. Interaction effect (result output for 2-way ANOVA analysis) (**B**)

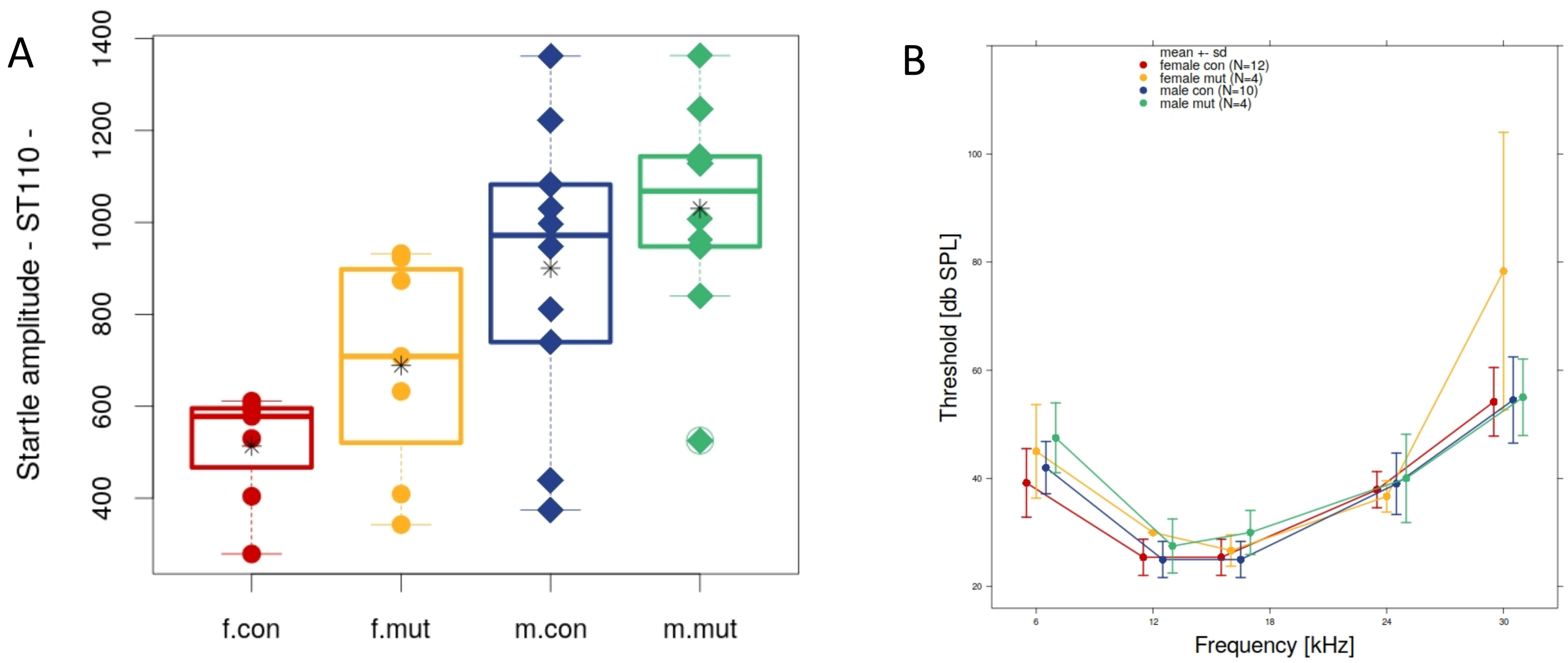

Fig. S5. **No differences in acoustic startle at 110 dB (A) or auditory brainstem response (B) to indicate differences in hearing sensitivity in *Tanc2*-disrupted mice.** F.con = female +/+, female mut = female -/-, m.con = male +/+, m.mut = male -/-

Disease Models & Mechanisms • Supplementary information

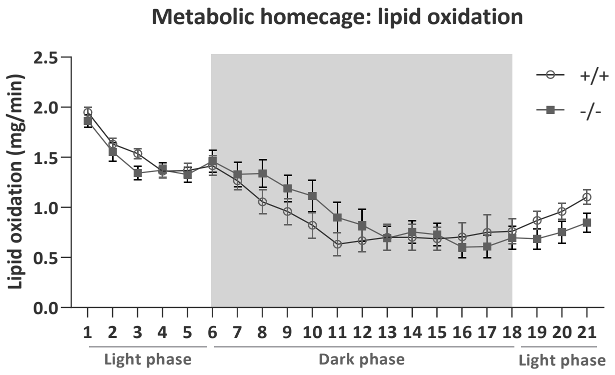

Fig. S6. No differences in lipid oxidation during indirect calorimetry analysis between homozygous *Tanc2*-disrupted mice (-/-) vs. Wildtype controls (+/+)

Table. S1. The testing age and number of mice used for each assay.

Assay	Age (weeks)	Number (n)			
		+/+		-/-	
		Males	Females	Males	Females
Open field	8	9	7	9	6
SHIRPA	9	7	5	7	5
Prepulse inhibition	10	10	7	10	7
Indirect calorimetry	11	10	8	10	7
Glucose tolerance test	13	10	7	10	7
DEXA	14	10	7	6	5
Auditory brainstem response	14	8	6	4	4
Clinical chemistry	16	10	7	8	7
Pathology	16	2	2	2	2

Disease Models & Mechanisms • Supplementary information
